# Emergency response for maritime sudden cardiac death: framework for pre-hospital rescue procedures and analysis of clinical characteristics

**DOI:** 10.3389/fcvm.2026.1700421

**Published:** 2026-05-07

**Authors:** Jinru Li, Mingqiu Hu, Tongyu Wang, Yanhao Xie, Yi Liu, Jian Yao, Xiangyu Yan, Haojun Fan

**Affiliations:** 1 School of Disaster and Emergency Medicine, Tianjin University, Tianjin, China; 2 The PRC Ministry of Education Engineering Research Center of Intelligent Technology for Healthcare, Wuxi, China; 3 The Key Laboratory of Medical Rescue Key Technology and Equipment, Ministry of Emergency Management, Tianjin, China; 4 Tianjin PKUCare CNOOC Hospital, Tianjin, China; 5 School of Artificial Intelligence and Computer Science, Jiangnan University, Wuxi, China

**Keywords:** maritime emergency response, offshore workers, pre-hospital emergency, sudden cardiac death, telemedicine

## Abstract

**Introduction:**

Sudden cardiac death (SCD) is a critical public health issue. Occupational workers face increased SCD risk, especially in high-risk maritime environments. Emergency care in these settings is limited by limited medical access and scarce pre-hospital emergency systems research.

**Methods:**

To address the risks associated with SCD incidents on offshore operational platforms, our study constructed a novel Telemedicine Maritime Assistance Service (TMAS) service framework for emergency response. This TMAS framework integrated real-time telemedicine supported by satellite communication and land-based hospital specialists, standardized on-site maritime emergency response protocols, and implemented coordinated aeromedical transport with geospatial-optimized helicopter deployment. We conducted a retrospective study of offshore workers in China's Bohai Bay region who experienced SCD between 2019 and 2023, and the resuscitation success rates were calculated. Additionally, a representative case study was included to document a real-world SCD emergency response scenario under the TMAS framework. To further assess long-term health implications, we analyzed surveillance data from annual health examinations to identify clinical characteristics and trends among the affected workers.

**Results:**

Seven male SCD cases (mean age 45.4 ± 5.8 years) occurred among offshore workers. A 42.86% resuscitation success rate was observed among these patients under the TMAS system. TMAS activation was contribute to successful aeromedical transport of approximately 2 h (35 nautical miles) in a representative 2022 case of SCD occurring in a 45-year-old worker at a Bohai Bay offshore drilling platform. Long-term continuous health surveillance was performed from 3 to 19 years (mean 15 years) in individuals who had experienced SCD. We identified five indicators with the highest overall abnormal detection rates, including low-density lipoprotein (100%), glycated hemoglobin (100%), glucose (57.1%), body mass index (57.1%), and systolic blood pressure (42.9%). Based on long-term trends in health surveillance data, the monitored indicators were classified into four categories, including long-term stable pattern (body mass index, white blood cell count, creatinine), downwards-trending in the 3 years preceding SCD pattern (low-density lipoprotein, high-density lipoprotein, glucose, etc.), upwards-trending in the 3 years preceding SCD pattern (lymphocytes, bilirubin), and uncertain trend pattern (diastolic blood pressure, systolic blood pressure, triglycerides, etc.).

**Conclusion:**

The TMAS-based emergency response mechanism was observed a higher success rate in SCD resuscitation among offshore workers. This study provides critical evidence supporting the optimization of emergency response strategies for occupational populations, indicating that long-term continuous health surveillance combined with an optimized maritime rescue mechanism should be established and reinforced.

## Introduction

Sudden cardiac death (SCD) is defined as an unexpected cessation of cardiac activity resulting in death within 1 h of symptom onset (1). SCD represents more than 50% of all cardiac-related deaths worldwide, with 4 to 5 million cases annually (2). China bears the highest global burden with 1.03 million annual cases, yet out-of-hospital survival rates remain critically low at approximately 4% (3, 4).

SCD increasingly affects younger and occupational populations (5). In China, working-age adults experience an 8%–12% annual increase in SCD incidence (5), with 41.5% of cases occurring in individuals aged 35 to 54 years (6). The Kailuan occupational cohort study reported a sixfold increase in cardiovascular disease incidence among young workers (18–44 years) from 2006 to 2008 to 2018–2020 (7). Surveys show that 25% of workers have undetected hypertension (8). Offshore work involves moderate-to-high occupational risk, with workers facing compounded hazards including remote environments, delayed medical access, and prolonged exposures (9, 10). Blue-collar workers have a significantly greater risk of SCD than white-collar workers (11). A systematic review confirmed that prolonged shift work increases cardiovascular disease incidence with an odds ratio of 2.1 (12). A 10-year retrospective cohort study revealed high prevalence rates of dyslipidemia (46.8%), hyperglycemia (23.1%), and obesity (41.2%) among offshore workers, with hypertension rates 1.8-fold higher than onshore counterparts (13). These findings are supported by 3D anthropometric data showing substantially greater obesity prevalence (42.1% vs. 28.9%) and adverse abdominal fat distribution patterns in offshore workers (14). Platform-based studies documented hyperuricemia detection rates of 34.4% to 38.6%, consistently higher among offshore workers (37.4%) than land-based workers (33.7%) (15). Despite these well-documented risks, systematic research on cardiovascular health in offshore oil workers remains disproportionately limited (13, 16).

Optimizing emergency care for offshore SCDs requires specialized protocols addressing geographical barriers and transport challenges. Previous studies have focused predominantly on land-based pre-hospital systems, with limited attention given to emergency telemedicine and transport processes in offshore settings (13, 16–18). This study aimed to establish and evaluate a Telemedicine Maritime Assistance Service (TMAS) framework for pre-hospital emergency response for maritime SCD on offshore oil platforms in China, and to explore the clinical characteristics of maritime SCD patients based on long-term health surveillance data.

## Methods

### Study setting

This study focused on offshore oil operation platforms in China's Bohai Bay, which are operated by the nation's largest offshore oil and gas producer. Frontline workers lived and worked exclusively on these artificial island-style drilling or production platforms, which were characterized by remote locations and unique maritime environments. The offshore drilling platform operated on a 4-week day–night shift rotation schedule, with 4 weeks of work followed by 4 weeks of leave. Workers typically spent between four and six hours daily at worksites, with potentially longer exposure during equipment maintenance or platform adjustment periods. During offshore operations, workers faced relatively high labor intensity, calorie-dense diets, and relatively limited recreational activities, which could increase the risk of acute cardiovascular events, such as SCD. Detailed descriptions of the medical personnel, supply configurations, and emergency response protocols for these platforms are provided in the Supplementary Material S1.

### Health surveillance and emergency response measures

#### Health surveillance based on annual health examinations

All offshore platform workers were required to complete annual comprehensive health examinations before commencing offshore assignments. The health examination data are systematically recorded in a dedicated database to enable continuous health monitoring and management. This protocol ensured that their physical conditions were suitable for offshore work requirements.

#### Telemedical maritime assistance service (TMAS)

For SCDs and other critical emergencies, an offshore emergency response system was established based on the TMAS combined with helicopter-based aeromedical transport. Rapid transportation and real-time teleconsultation, could be rapidly activated in times of emergency need, achieving seamless integration of sea‒land‒air medical resources. This ensured that patients received both timely advanced interventions and continuous specialist guidance, significantly improving offshore rescue efficacy and survival outcomes.

The TMAS was a comprehensive three-dimensional emergency response system designed to deliver rapid medical intervention for offshore workers experiencing acute conditions such as SCD. This integrated model leveraged sea‒land‒air information networks to coordinate multistage rescue efforts (Supplementary Figure S2). The protocol commenced with immediate on-platform assessment and cardiopulmonary resuscitation by stationed physicians, followed by synchronized mobilization of aerial transport resources and shore-based emergency teams for direct hospital transport. Continuous real-time telemedicine support from land-based specialists ensured uninterrupted clinical guidance during transit. This optimized system achieved the dual objectives of minimizing time–critical transport delays while maximizing treatment efficacy throughout the care continuum.

#### Emergency response process framework and TMAS protocol

**Figure 1:**
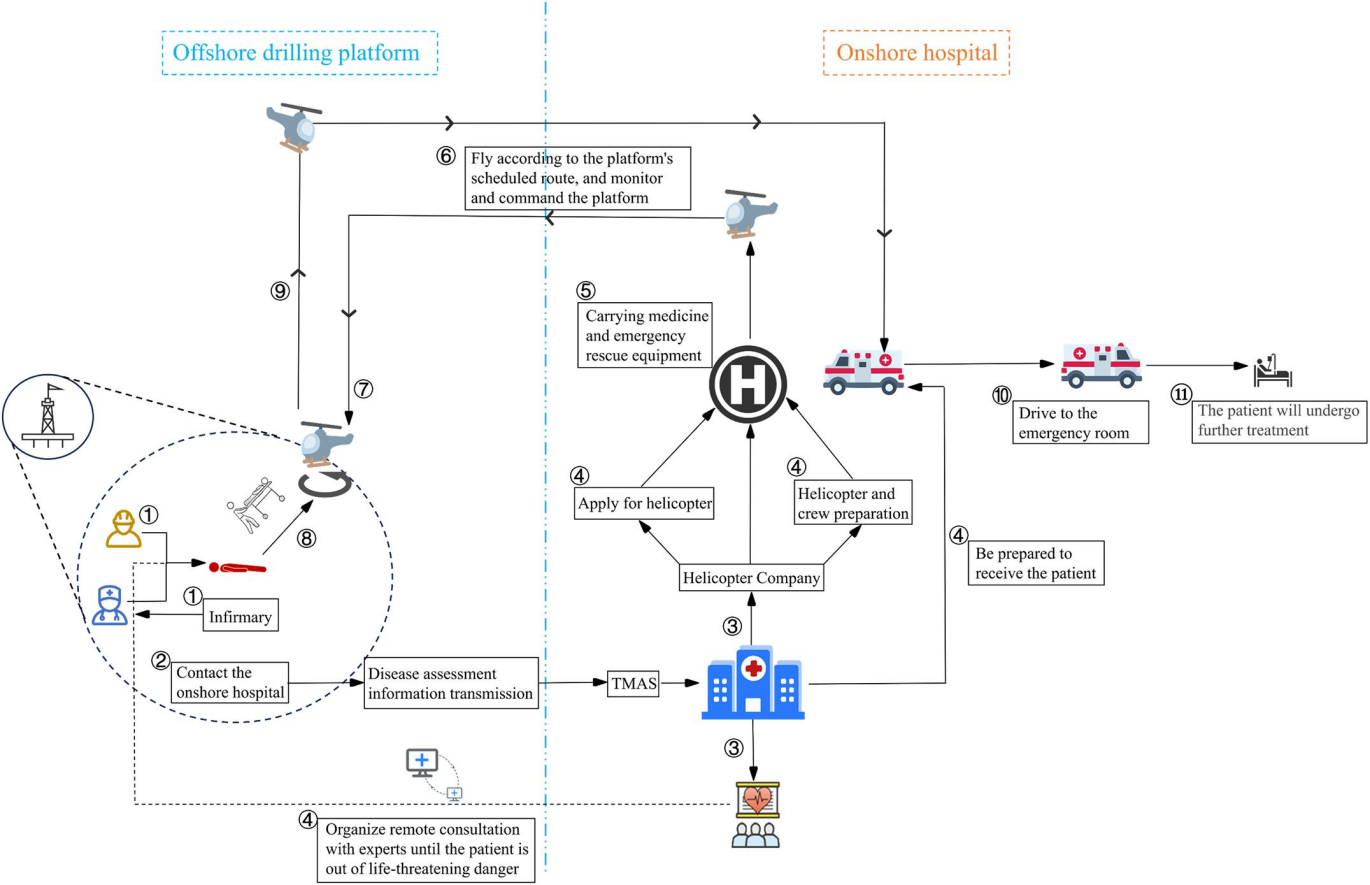
Overall framework for emergency response process.

In the TMAS, the emergency response process after SCD occurrence was structured into five major procedures (Figure 1). This framework has been implemented as part of the standard TMAS protocol for all offshore platforms in the Bohai Bay region since 2019.

##### Immediate response and initial treatment

1. Finding and reporting: When a worker experiences SCD on the offshore platform, coworkers promptly initiate emergency protocols, and simultaneously alert the on-site medical officer and the TMAS center.
2. Initial treatment: The platform doctor arrives at the scene quickly to perform cardiopulmonary resuscitation (CPR) and other initial treatment measures, while preparing for patient transport.

##### Information transmission and decision-making

1. Telemedical information transmission: The platform physician performs an initial patient assessment and transmits critical medical data, including electrocardiogram (ECG) readings and a patient summary, to the onshore TMAS center and land-based hospital through the satellite telemedicine system.
2. Consultation and decision: Upon receiving the transmitted data, the land-based hospital immediately convenes a specialist team for remote consultation. The medical team continues monitoring and treating the patient until life-threatening conditions stabilized, while simultaneously activating the TMAS emergency response.

##### Aerial rescue preparation

1. Airspace application: The helicopter operating company immediately requests airspace clearance from aviation authorities to secure an unobstructed flight path for the rescue mission.
2. Helicopter and crew preparation: The flight crew conducts preflight checks, inspecting the helicopter's operational status and loading essential medical supplies and equipment.
3. Onshore coordination: The receiving hospital readies the landing zone to ensure safe aircraft arrival, while the emergency medical team and ambulance crew remain on standby, and are fully prepared to receive and manage the patient immediately upon landing.

##### Aerial transportation guided by land-based experts

1. Takeoff and arrival at platform: The helicopter takes off and flies to the offshore platform according to the predetermined route. Upon arrival, platform doctors quickly transported the patient to the helicopter.
2. Flight process: During transit, doctors on the helicopter maintain continuous treatment and monitoring to stabilize the patient's condition, while remaining in constant communication with land-based medical specialists for guidance.

##### Arrival at land-based hospital and subsequent treatment

1. Landing and transport: After landing on the helicopter pad of the land-based hospital, doctors quickly transport a waiting ambulance for rapid ground transport to the emergency room.
2. Emergency treatment: The emergency room immediately initiates the priority care pathway. For patients with acute cardiopulmonary failure, clinicians rapidly initiated extracorporeal membrane oxygenation (ECMO) to provide vital organ support
3. Follow-up observation and treatment: After the patient's condition stabilized, the patient was sent to the cardiology intensive care unit for further observation and treatment.

This emergency response framework has demonstrated critical efficacy in managing SCD cases on offshore platforms. To systematically evaluate the operational protocol, we conducted a comprehensive retrospective analysis of historical medical transport records and selected a representative case for detailed process mapping. Using temporal node data extracted from each rescue phase, we performed an event timeline analysis to assess the framework's operational efficacy.

### Statistical analysis of health surveillance data among SCD workers

#### Data collection

The study data were obtained from the annual health examination records of workers who experienced SCD between 2019 and 2023.

1. Basic information: This information includes details such as the time, encrypted ID numbers, gender, age, occupational category, unit, time of SCD, and SCD diagnosis.
2. Health examination indicators: These information includes routine blood tests (white blood cell count, red blood cell count, hemoglobin, neutrophil percentage, lymphocyte percentage, etc.), biochemical tests (glucose, triglycerides, alanine aminotransferase, aspartate aminotransferase, albumin, globulin, the albumin-globulin ratio, total bilirubin, blood urea nitrogen, creatinine, uric acid, total cholesterol, high-density lipoprotein, low-density lipoprotein, etc.), and internal medicine examinations (systolic blood pressure, diastolic blood pressure, body mass index, etc.).

#### Data processing

To ensure data reliability and accuracy, we averaged multiple health examination entries within the same year to eliminate duplicates, as these reports are typically conducted annually. Additionally, we standardized the measurement units across different years for consistency.

Regarding missing data, we implemented a rigorous multiple imputation procedure using Multiple Imputation by Chained Equations (MICE) for variables with sporadic missing values. Subsequent models were fitted to the multiply imputed datasets, and results were pooled using Rubin's rules to obtain final estimates.

#### Descriptive statistical analysis

We conducted descriptive statistical analysis on the demographic and clinical characteristics of offshore workers who experienced SCD from 2019 to 2023, The analysis included distributions of age, sex, and other demographic statistics.

For each indicator, we computed the mean and standard deviation for each worker individually via SPSS software. These values were subsequently aggregated to produce the overall mean and standard deviation for all workers' descriptive statistics. Furthermore, we divided all of the patients into two groups based on resuscitation outcomes (successful vs. unsuccessful) and recalculated the mean and standard deviation for each group.

We ascertained the resuscitation outcomes through a systematic review of platform medical records, counted the number of successful resuscitations, and calculated the resuscitation success rate. Clinical reference ranges from contemporaneous health examinations define abnormal parameters, where values exceeding established norms are flagged as clinically significant. The corresponding abnormality detection rate was calculated as follows: Abnormality detection rate = (Cases with abnormal values/Total evaluated workers) × 100%.

Using the collected clinical examination data, we plotted line charts to monitor long-term trends in relevant indicators before SCD. For each indicator, 95% confidence intervals (CIs) were calculated for means and proportions where applicable to enhance the rigor of the analysis. All the statistical analyses were performed via IBM SPSS software, version 27 (IBM Corp., Armonk, NY, USA).

#### Ethical issues

The study was reviewed and approved by the institutional review board of Tianjin University Medical Ethics Committee (approval ID: TJUE-2024-434) and the Ethics Committee of PKU Care CNOOC Hospital. The requirement for informed consent of participants was waived owing to the use of deidentified data obtained as part of routine health check-ups.

## Results

### Representative TMAS process

**Figure 2:**
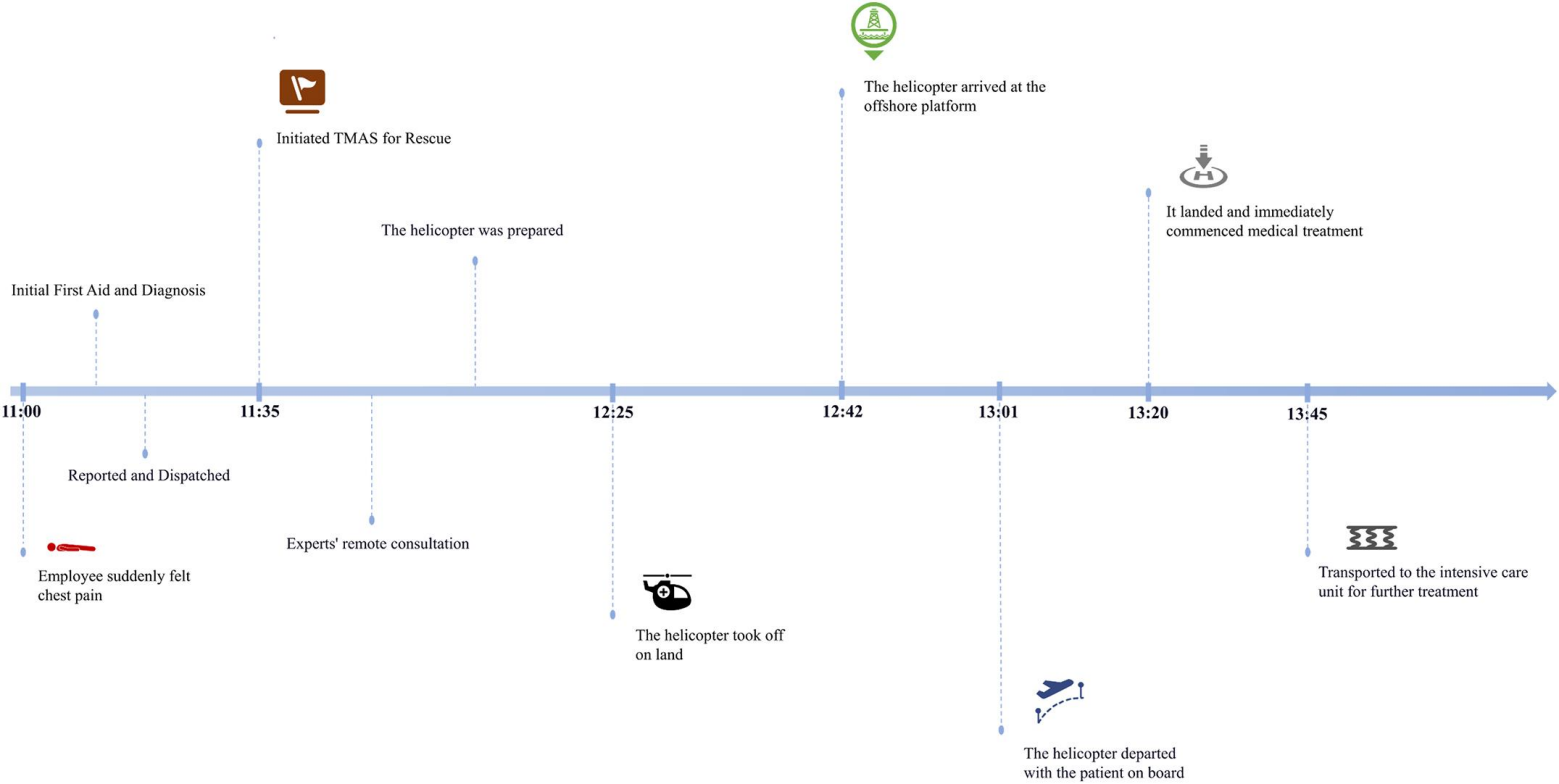
Timeline of offshore SCD worker transport by helicopter.

The TMAS process was illustrated through its application in a 2022 maritime emergency. A worker on an offshore platform experienced SCD during midday operations. Following the TMAS protocol, the patient received on-site CPR, telemedicine-guided management, and helicopter transport to a land-based hospital. From symptom onset to hospital arrival, the rescue covered approximately 35 nautical miles and took just over 2 h. Coronary angiography confirmed right coronary artery occlusion, and the patient was successfully resuscitated after stent implantation. Figure 2 presents the overall timeline of this TMAS process. A detailed minute-by-minute breakdown of the rescue sequence is provided in the Supplementary Materials.

### Basic information of patients with SCD

From 2019 to 2023, seven male workers experienced SCD on offshore platforms, with ages ranging from 40‒57 years (mean age 45.43 ± 5.83 years). All patients received immediate on-site resuscitation, resulting in three successful resuscitations and four unsuccessful cases. The successfully resuscitated group had a mean age of 46 years (46 ± 7.21), whereas the mean age of the non-resuscitated group was 45 years (45 ± 5.72). The resuscitation success rate for offshore platform workers was 42.86% (3/7). Review of platform medical records and rescue documentation confirmed that all SCD events were attributable to acute myocardial infarction, consistent with ischemic heart disease.

All of the patients received the complete TMAS protocol, including immediate on-site resuscitation by platform physicians, real-time telemedicine consultation with land-based specialists, and coordinated helicopter aeromedical transport to receiving hospitals. Platform medical records confirmed that each phase of the TMAS protocol was completed for all of the patients.

Workers who experienced SCD came from diverse occupational backgrounds, including two operators, one scaffolder, one instrumentation and control engineer, one electrician, and one ship captain (Supplementary Figure S3). All affected workers received immediate on-site treatment, with three successfully resuscitated workers and four not resuscitating workers. The workers had medical records spanning durations ranging from 3 to 19 years, with a mean of 15 years. The number of health examination reports varied from 3 to 32, with an average of 21 reports.

### Clinical characteristics

#### Laboratory test characteristics of health examinations

A total of 25 health examination indicators were included in the analysis. Among them, the overall average values of the five indicators were higher than the normal range. These five indicators were systolic blood pressure (mean 144.87 ± 31.34 mmHg, 95% CI: 115.62–174.12), body mass index (mean 23.9 ± 2.52 kg/m², 95% CI: 21.57–26.23), glucose (mean 6.37 ± 0.82 mmol/L, 95% CI: 5.62–7.12), low-density lipoprotein (mean 3.86 ± 0.43 mmol/L, 95% CI: 3.46–4.26), and glycated hemoglobin (mean 6.5 ± 0.95%, 95% CI: 4.57–8.43).

The abnormal detection rates for the above indicators were low-density lipoprotein (100%, 7/7), glycated hemoglobin (100%, 3/3), glucose (57.14%, 4/7), body mass index (57.14%, 4/7), and systolic blood pressure (42.86%, 3/7) (Table 1).

**Table 1:** Laboratory test characteristics of the seven patients.

Indicators	Reference range	Overall	Successfully resuscitated group				Non-resuscitated group				
		Value (x¯ ± SD) [95% CI]	A	B	C	Average value	D	E	F	G	Average value
Heart rate [bpm]	60–100	80.06 ± 8.56 [72.50–87.62]	77.82 ± 5.32	—^b^	74.75 ± 3.81	76.29 ± 2.17	97.00 ± 13.26	—^b^	74.44 ± 2.70	—^b^	76.29 ± 15.95
Diastolic blood pressure [mmHg]	60–89	88.37 ± 6.83 [82.13–94.61]	84.74 ± 4.64	77.22 ± 9.80	100.47 ± 14.37	87.48 ± 11.86	92.16 ± 6.14	83.16 ± 6.92	86.71 ± 4.75	95.00 ± 13.23	87.48 ± 5.33
Systolic blood pressure [mmHg]	90–139	144.87 ± 31.34 [115.62–174.12]	125.47 ± 12.03	104.17 ± 23.86	219.59 ± 286.7	149.74 ± 61.42	141.05 ± 9.56	138.47 ± 8.97	132.43 ± 11.61	148.00 ± 17.00	149.74 ± 6.45
Body mass index [kg/m²]	18.5–24.9	23.90 ± 2.52 [21.57–26.23]	22.10 ± 0.94	19.27 ± 0.54	26.83 ± 1.29	22.73 ± 3.82	27.78 ± 1.67	24.24 ± 1.17	24.80 ± 0.99	23.46 ± 0.11	22.73 ± 1.89
Alanine aminotransferase (ALT) [U/L]	10–40	34.60 ± 21.00 [15.78–53.42]	37.00 ± 14.70	9.59 ± 2.48	57.44 ± 21.52	34.68 ± 24.01	57.00 ± 36.94	60.66 ± 21.96	11.79 ± 2.94	8.67 ± 4.04	34.68 ± 28.13
Aspartate aminotransferase (AST) [U/L]	10–40	27.28 ± 11.86 [16.52–38.04]	21.22 ± 3.29	16.82 ± 2.94	36.88 ± 8.59	24.97 ± 10.54	43.92 ± 22.31	44.56 ± 16.29	15.54 ± 3.04	14.33 ± 1.15	24.97 ± 16.93
Albumin [g/L]	35–55	47.46 ± 1.53 [46.04–48.88]	47.98 ± 2.28	46.58 ± 2.05	48.73 ± 2.37	47.76 ± 1.09	48.00 ± 2.05	44.97 ± 11.63	49.98 ± 1.55	45.67 ± 1.15	47.76 ± 2.29
Globulin [g/L]	20–30	28.83 ± 1.69 [27.27–30.39]	27.85 ± 1.54	30.71 ± 3.56	28.71 ± 2.61	29.09 ± 1.47	—^b^	31.63 ± 2.92	26.92 ± 2.35	26.87 ± 1.65	29.09 ± 2.73
Albumin/globulin ratio	1.1–2.5	1.66 ± 0.11 [1.56–1.76]	1.72 ± 0.13	1.52 ± 0.18	1.71 ± 0.23	1.65 ± 0.11	1.65 ± 0.18	1.50 ± 0.17	1.87 ± 0.18	1.70 ± 0.12	1.65 ± 0.15
Total bilirubin [μmol/L]	3.4–20.5	13.64 ± 2.85 [11.00–16.28]	10.16 ± 2.23	10.12 ± 3.88	17.85 ± 26.51	12.71 ± 4.45	13.84 ± 4.03	18.21 ± 3.79	12.56 ± 3.18	13.67 ± 4.55	12.71 ± 2.49
Urea nitrogen [mmol/L]	2.5–7.5	5.40 ± 0.87 [4.60–6.20]	5.11 ± 0.87	5.50 ± 0.81	5.34 ± 1.31	5.32 ± 0.20	4.17 ± 0.89	4.98 ± 1.48	5.33 ± 0.88	7.45 ± 1.07	5.32 ± 1.40
Creatinine [mmol/L]	53–106	67.12 ± 11.59 [56.38–77.86]	88.22 ± 5.79	69.69 ± 5.26	61.83 ± 8.69	73.25 ± 13.55	60.75 ± 5.49	50.18 ± 4.72	77.20 ± 3.77	55.80 ± 6.39	73.25 ± 11.64
Uric acid [μmol/L]	149–416	354.22 ± 61.47 [297.27–411.17]	355.31 ± 52.20	265.57 ± 21.12	341.21 ± 55.52	320.70 ± 48.26	279.13 ± 43.09	447.00 ± 67.36	406.80 ± 28.64	418.00 ± 158.50	320.70 ± 74.36
Glucose [mmol/L]	3.9–6.1	6.37 ± 0.82 [5.61–7.13]	5.73 ± 0.43	5.84 ± 1.10	7.73 ± 2.06	6.43 ± 1.12	7.69 ± 0.91	6.26 ± 1.04	5.69 ± 0.47	5.60 ± 0.44	6.43 ± 0.97
Total cholesterol [mmol/L]	<5.2	5.45 ± 0.62 [4.88–6.02]	5.36 ± 0.54	5.50 ± 0.54	4.76 ± 0.52	5.21 ± 0.39	5.30 ± 0.75	6.72 ± 0.90	6.03 ± 0.57	4.70 ± 0.39	5.21 ± 0.88
Triglycerides [mmol/L]	<1.7	1.46 ± 0.44 [1.05–1.87]	0.97 ± 0.34	0.94 ± 0.22	1.97 ± 0.65	1.29 ± 0.59	1.67 ± 0.33	2.28 ± 0.50	1.32 ± 0.24	1.25 ± 0.38	1.29 ± 0.47
High-density lipoprotein (HDL) [mmol/L]	> 1.0	1.31 ± 0.16 [1.16–1.46]	1.30 ± 0.18	1.41 ± 0.14	1.24^a^	1.32 ± 0.09	1.36 ± 0.44	0.98 ± 0.10	1.56 ± 0.17	—^b^	1.32 ± 0.29
Low-density lipoprotein (LDL) [mmol/L]	<3.4	3.86 ± 0.43 [3.46–4.26]	3.76 ± 0.39	4.05 ± 0.35	3.13^a^	3.65 ± 0.47	3.73 ± 0.85	4.62 ± 0.90	4.11 ± 0.56	—^b^	3.65 ± 0.45
Glycated hemoglobin (HbA1c) [%]	4∼6	6.50 ± 0.95 [4.80–8.20]	—^b^	5.56 ± 0.09	—^b^	—^b^	7.68 ± 0.78	7.19 ± 0.56	—^b^	—^b^	5.56 ± 0.35
White blood cells (WBC) [×10^9^/L]	4.0–10.0	7.46 ± 1.81 [5.79–9.13]	5.69 ± 0.87	5.58 ± 0.98	8.80 ± 0.80	6.69 ± 1.83	9.17 ± 0.81	10.24 ± 1.55	5.08 ± 1.20	8.43 ± 1.52	6.69 ± 2.23
Neutrophil percentage [%]	40–75	59.21 ± 4.28 [55.28–63.14]	51.92 ± 4.32	57.87 ± 8.46	60.75 ± 3.74	56.85 ± 4.50	62.09 ± 4.19	56.48 ± 5.76	60.28 ± 7.57	67.40 ± 4.61	56.85 ± 4.54
Lymphocyte percentage [%]	20–50	31.82 ± 4.96 [27.24–36.40]	38.72 ± 4.59	34.50 ± 7.76	31.38 ± 2.77	34.87 ± 3.68	27.83 ± 4.35	35.55 ± 4.76	29.80 ± 8.16	21.87 ± 3.35	34.87 ± 5.64
Red blood cells (RBC) [×10^12^/L]	4.5–5.9	5.05 ± 0.22 [4.85–5.25]	4.89 ± 0.12	5.09 ± 0.20	5.21 ± 0.25	5.06 ± 0.16	5.35 ± 0.26	5.29 ± 0.30	4.86 ± 0.21	4.68 ± 0.07	5.06 ± 0.33
Hemoglobin [g/L]	130–180	157.60 ± 5.69 [152.34–162.86]	152.74 ± 4.52	155.50 ± 7.85	161.93 ± 8.53	156.72 ± 4.72	169.11 ± 7.17	160.63 ± 7.34	154.14 ± 8.32	150.00 ± 4.36	156.72 ± 8.33
Specific gravity of urine	1.005–1.030	1.03 ± 0.02 [1.01–1.05]	1.02 ± 0.01	1.02 ± 0.01	1.02 ± 0.01	1.02 ± 0.00	1.09 ± 0.01	1.02 ± 0.00	1.02 ± 0.01	1.02 ± 0.00	1.02 ± 0.04
Urine pH	4.5–8.0	5.91 ± 0.28 [5.65–6.17]	5.86 ± 0.35	5.50 ± 0.46	5.90 ± 0.46	5.70 ± 0.28	5.66^a^	6.08 ± 0.58	6.20 ± 0.89	6.33 ± 0.58	5.70 ± 0.29

a This individual did not undergo testing for this indicator.

b This indicator was only tested once, therefore, the calculation of the average and standard deviation was not possible.

#### Long-term trends in health examination indicators

Among the 25 indicators under long-term monitoring, three indicators (whole blood viscosity at shear rates of 1 s⁻¹ and 5 s⁻¹, and urea) were excluded from the trend line chart presentation due to insufficient sample size (annual average participants <4) and poor data continuity (effective consecutive monitoring duration <3 years), which failed to meet the requirements for long-term trend observation. Consequently, the line charts presented consequently display only the remaining 22 indicators that met the inclusion criteria.

Based on long-term trend analysis, the indicators were categorized into four distinct categories. The first category comprises long-term stable indicators, characterized by minimal fluctuations over the past decade. Notably, BMI exhibited persistent abnormal stability, remaining consistently outside normal ranges throughout the observation period. This category comprises 3 indicators, including the body mass index (abnormal detection rate: 57.14%, 4/7), white blood cell count (71.43%, 5/7), and creatinine (57.14%, 4/7) (Supplementary Figure S4).

**Figure 3:**
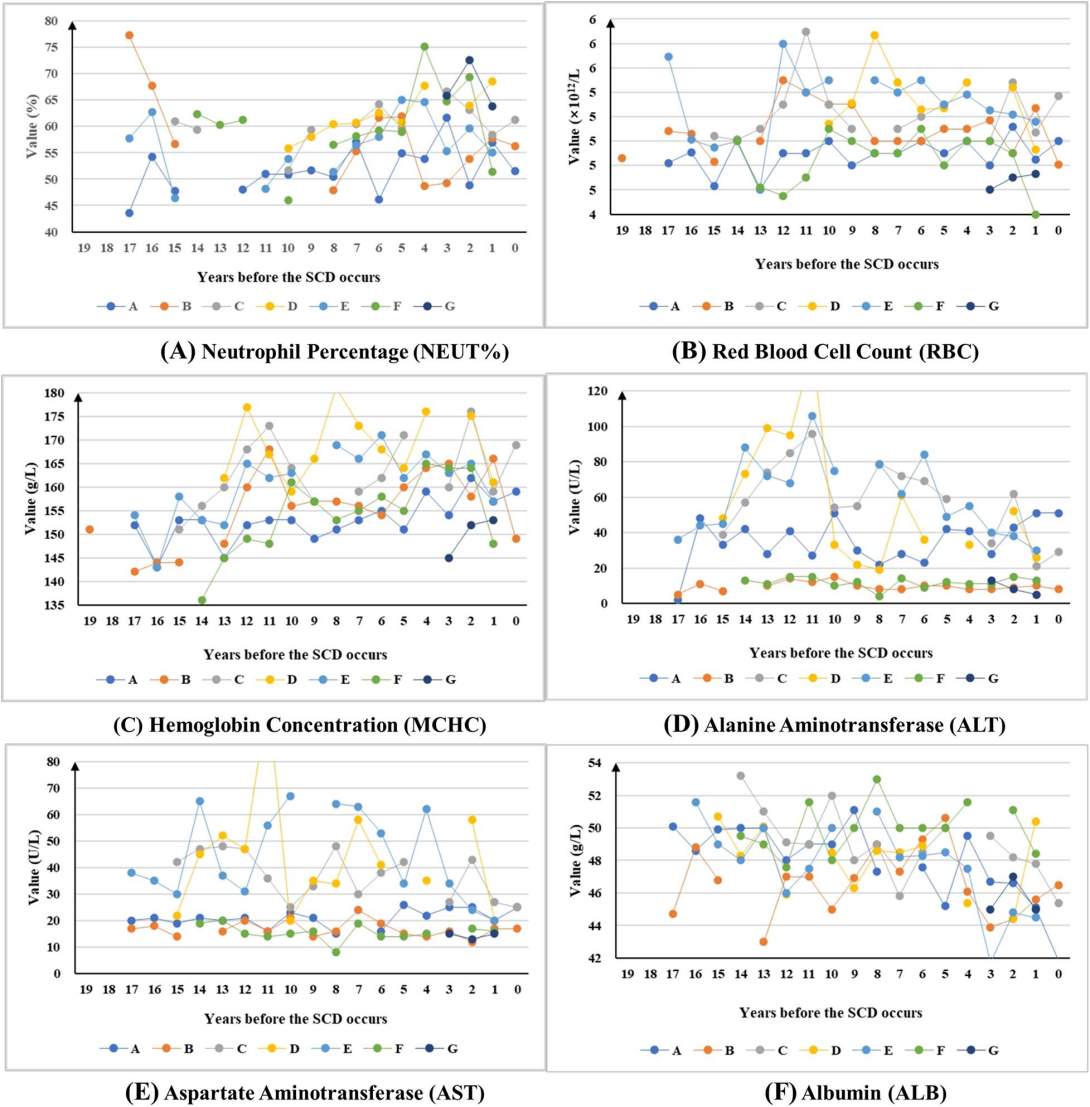
The long-term trends of indicators show downwards-trending patterns in the 3 years preceding SCD.

The second category of indicators has experienced significant fluctuations over an extended period and showed a clear downwards trend in the three years pre-SCD period. This category comprised 12 indicators, including the neutrophil percentage (71.43%, 5/7), red blood cell count (57.14%, 4/7), hemoglobin concentration (85.71%, 6/7), alanine aminotransferase (57.14%, 4/7), aspartate aminotransferase (42.86%, 3/7), albumin (85.71%, 6/7), urea nitrogen (71.43%, 5/7), uric acid (71.43%, 5/7), glucose (85.71%, 6/7), total cholesterol (85.71%, 6/7), high-density lipoprotein cholesterol (100%, 5/5), and low-density lipoprotein cholesterol (100%, 5/5) (Figure 3).

**Figure:**
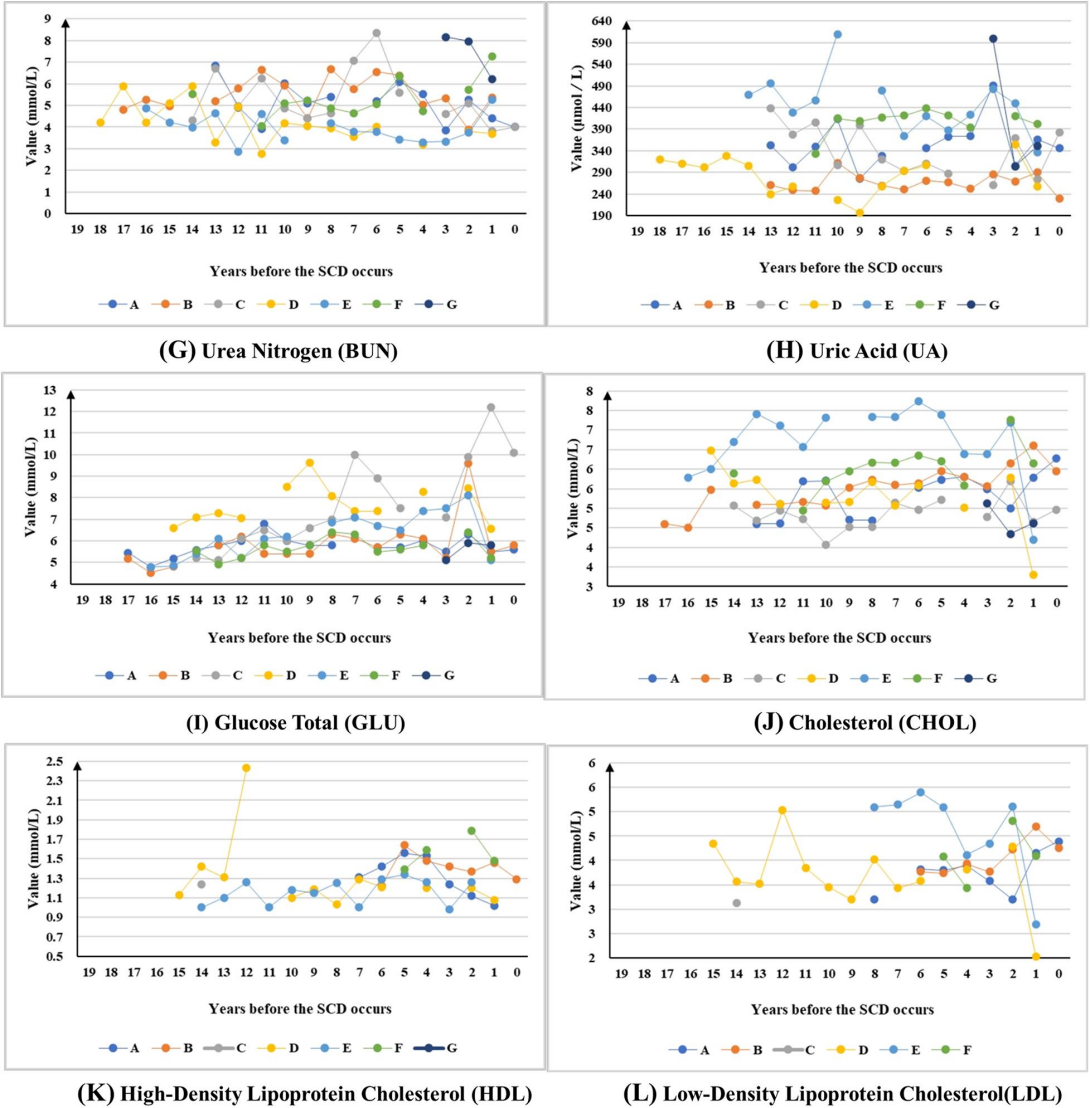


**Figure 4:**
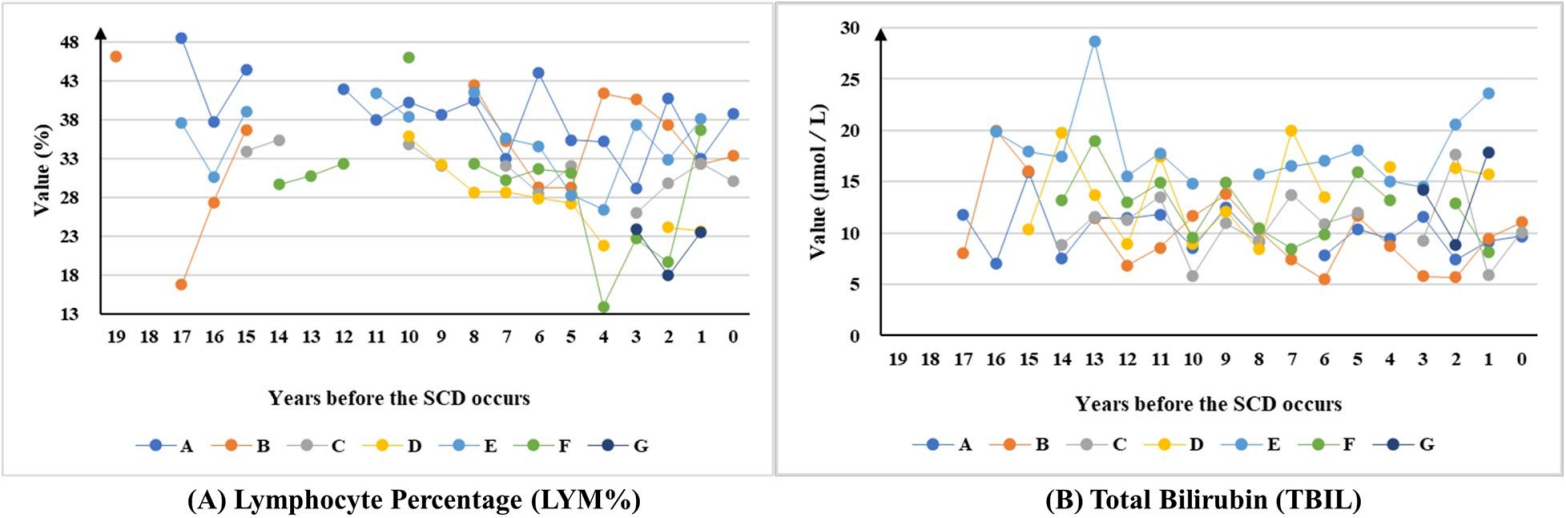
The long-term trends of indicators show upwards-trending patterns in the 3 years preceding SCD.

The third category of indicators also exhibits significant fluctuations over a long period but shows a clear upwards trend in the three years before the SCD. This category comprised 2 indicators, including the lymphocyte percentage (71.43%, 5/7) and total bilirubin (42.86%, 3/7) (Figure 4).

The fourth category of indicators is the uncertain trend type. This category included indicators that showed neither a clear upwards nor downwards trend over the long term, nor did they exhibit any consistent pattern in the three years before the SCD. This category comprises 5 indicators, including the diastolic blood pressure (100%, 7/7), systolic blood pressure (100%, 7/7), globulin (66.67%, 4/6), A/G ratio (57.14%, 4/7), and triglycerides (57.14%, 4/7) (Supplementary Figure S5).

## Discussion

### Key findings

Prehospital resuscitation success rates in China remain critically low, with a survival rate of only 1.3% (1). The TMAS in our study was contributed to a 42.86% resuscitation success rate among all of the patients. This observed rate is higher than conventional protocols and exceeds international benchmarks, including rates of 10.7–33.7% in the US (19), 8.5–30.2% in Europe (20), and 12.5–28.4% in Japan (21). When compared with other maritime or remote occupational cohorts, this observed success rate is notably higher than the 8.5–30.2% reported for out-of-hospital cardiac arrest in European prehospital systems (20) and the 10.7–33.7% in US settings (19). However, direct comparisons should be interpreted with caution due to differences in case definition, transport logistics, and healthcare system contexts across studies.

Land pre-hospital emergency systems (e.g., Shanghai) demonstrate median operational intervals of 1.5 min (call processing), 8.2 min (ambulance arrival), and 8.7 min (advanced life support initiation), with a 26.4 min prehospital care continuum (22). Historical cohorts show sequential phases, including field stabilization, patient loading (3.2 min), CPR during evacuation, and hospital transport (18–22 min) (23). Conversely, the maritime rescue involves a complex, multistage operational process, including on-site SCD identification, telemedical emergency reporting, aerial deployment (airspace clearance and helicopter dispatch), ground preparations and specialized medical transport. This systematic comparison highlights how terrestrial systems prioritize rapid sequential response, while maritime rescue requires prolonged, coordinated multiagency interventions owing to environmental and logistical constraints.

This study identified distinct pre-SCD indicator dynamics. Multiple indicators, particularly glucose (GLU), high-density lipoprotein (HDL), low-density lipoprotein (LDL), and uric acid (UA), showed significant long-term fluctuations and consistent downward trends in the three years preceding SCD. In contrast, lymphocyte percentage (LYM%) and total bilirubin (TBIL) exhibited upward trends during the same period. These observed patterns may act synergistically through multiple pathophysiological pathways to destabilize atherosclerotic plaques, although causal inference is limited by the observational study design.

The potential biological mechanisms underlying these observations warrant consideration. Specifically, glycemic variability induces endothelial oxidative stress, which not only impairs myocardial function but also accelerates the progression of cardiac damage (24–26). This mechanism has been independently associated with both the presence and severity of cardiovascular disease in diabetic populations (27). The observed downward trends in lipoproteins (HDL and LDL) and glucose in the three years preceding SCD may reflect a convergence of metabolic dysregulation prior to the acute event, rather than a protective phenomenon. The concurrent hemodynamic stress arising from blood pressure fluctuations, which is particularly pronounced in shift workers experiencing circadian disruption, appears to accelerate plaque vulnerability through mechanical forces acting on vascular walls (11, 13, 28). Furthermore, metabolic–hemodynamic interactions create a high-risk profile characterized by triglyceride–glucose index elevation, compounding overall cardiovascular risk (29). Emerging evidence highlights the prognostic value of inflammatory biomarkers in cardiovascular risk assessment. The neutrophil percentage–albumin ratio and neutrophil–lymphocyte ratio independently predict mortality in heart failure patients (30), whereas elevated bilirubin levels (consistent with our TBIL trend) demonstrate established prognostic utility in acute coronary syndrome patients (31). The consistent three-year pre-event biomarker patterns observed in this small cohort of offshore workers preceding SCD suggest a potential window for targeted intervention, but these findings require validation in larger prospective studies.

This study has several limitations. First, the single-region sampling from Bohai Bay limits the generalizability of our findings to other offshore or maritime settings. Second, the small sample size constrained statistical power and precluded subgroup analyses. Third, although the study included long-term health surveillance data spanning up to 19 years, the retrospective case series design limits causal inference regarding the observed biomarker trends. Fourth, the routine health examination database did not include physiological or behavioral data, such as diet, physical activity, or sleep patterns. Future studies should include larger sample sizes, multicenter collaboration, and collection of behavioral data to better characterize SCD risk in offshore workers.

## Conclusion

Long-term health surveillance (mean 15 years) identified five high-risk indicators (LDL and HbA1c at 100% abnormality) and long-term trends of the four indicators preceding SCD, including stable (BMI, WBC, Cr), downwards-trending in the 3 years preceding SCD (LDL, HDL, GLU, etc.), upwards-trending in the 3 years preceding SCD ((LYM%, TBIL), and uncertain trends (DBP, SBP, TG, etc.). The TMAS-supported emergency response system contributed to favorable resuscitation outcomes for SCD cases among maritime workers in this cohort. This study provides essential evidence for the development of occupational emergency protocols, highlighting the necessity of implementing sustained health monitoring systems and enhancing maritime medical rescue operations. Further prospective studies with larger sample sizes and control groups are needed to confirm these findings.

## Data Availability

The data analyzed in this study is subject to the following licenses/restrictions: The datasets generated and analyzed during the current study are available from the corresponding author upon reasonable request. Requests to access these datasets should be directed to Xiangyu Yan, yanxiangyu@tju.edu.cn.
