# Supplementary material for: Emergency response for maritime sudden cardiac death: framework for pre-hospital rescue procedures and analysis of clinical characteristics
Source: Front Cardiovasc Med. 2026 May 7;13:1700421. doi: 10.3389/fcvm.2026.1700421 (PMC13189821; doi:10.3389/fcvm.2026.1700421)
Supplement: Supplementary file 1 [file Datasheet1.docx]

**Medical personnel and supply configurations for offshore platforms**

Every offshore platform maintained a dedicated infirmary staffed by at least one permanently stationed physician responsible for daily health consultations, preliminary diagnoses, and medical treatment. These offshore medical practitioners were required to hold valid practicing physician certification and general practitioner training credentials, complemented by a minimum of three years of clinical experience. Essential qualifications included specialized training in maritime medical rescue, specifically advanced cardiac life support (ACLS) and international trauma life support (ITLS) certifications. Furthermore, mandatory biennial standardized offshore emergency training and periodic rescue drills were implemented to ensure continuous skill enhancement.

Offshore operational platforms maintain four categories of essential medical emergency supplies: (1) emergency medications, including epinephrine injections, nitroglycerin tablets, and fast-acting antianginal agents; (2) respiratory support devices, including bag-valve masks, oropharyngeal airways, and portable ventilators; (3) oxygen supply systems, including high-pressure oxygen regulators and compressed oxygen cylinders; and (4) auxiliary equipment, including folding stretchers, electrotherapy devices, and UV sterilization lamps. Furthermore, all platform infirmaries displayed standardized emergency protocol flowcharts in visible areas, facilitating rapid and accurate medical responses (see Supplementary Figure S1).

| 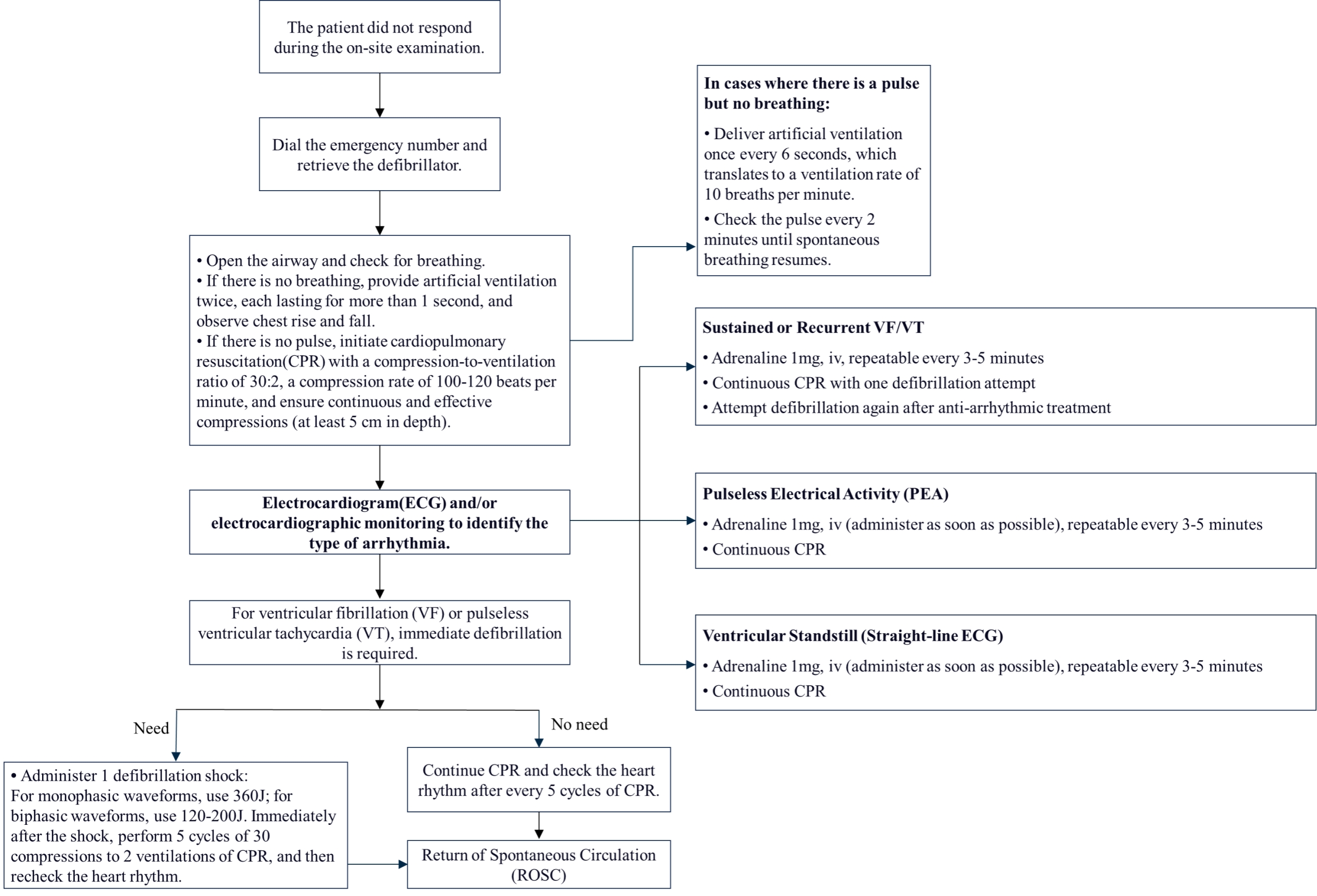 |
| --- |
| **Supplementary Figure S1.** **Emergency protocol flowchart.** |

| 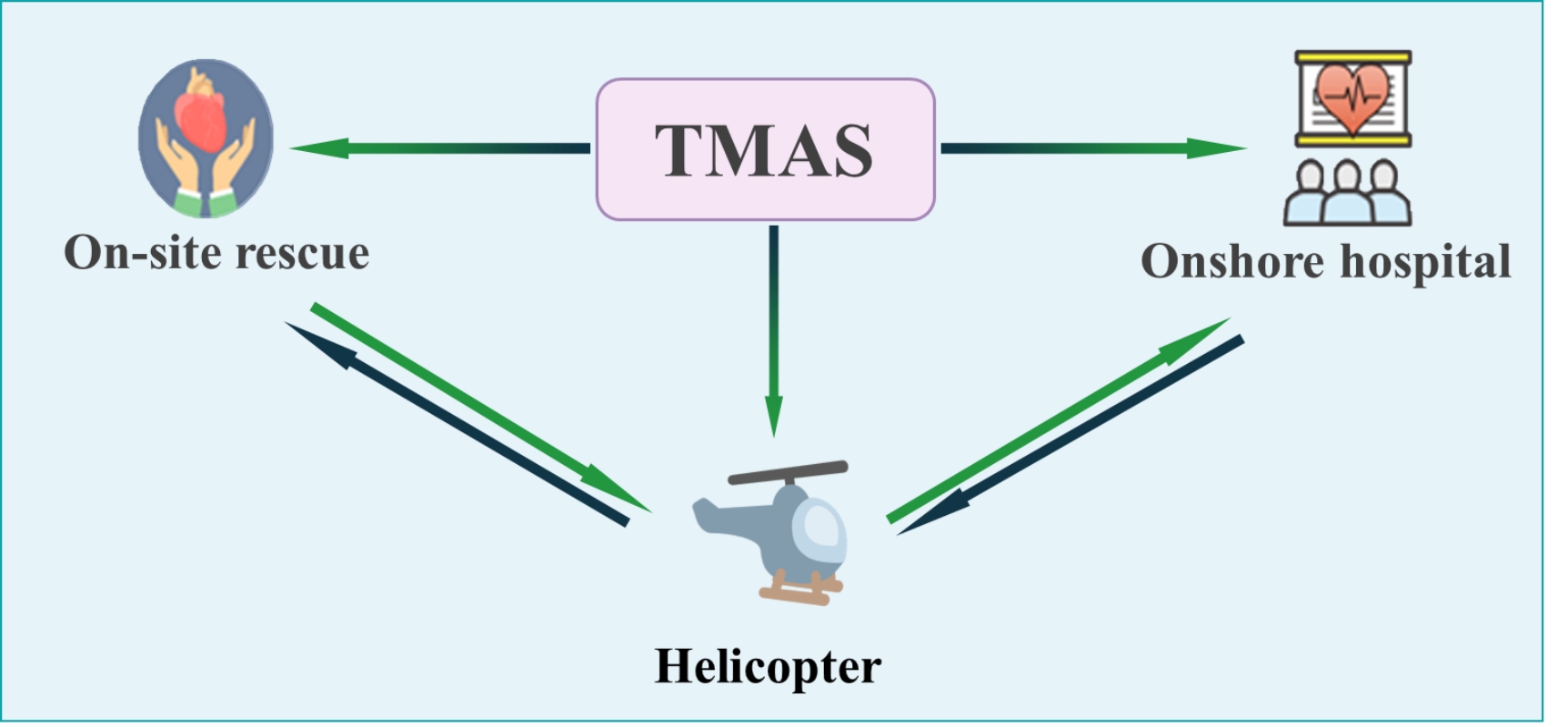  **Supplementary Figure S2. Emergency flowchart for**  **sea‒land‒air medical rescue management.** |
| --- |

| **Supplementary Figure S3. Summary of medical examination data reports for cardiac arrest patients** |
| --- |

| 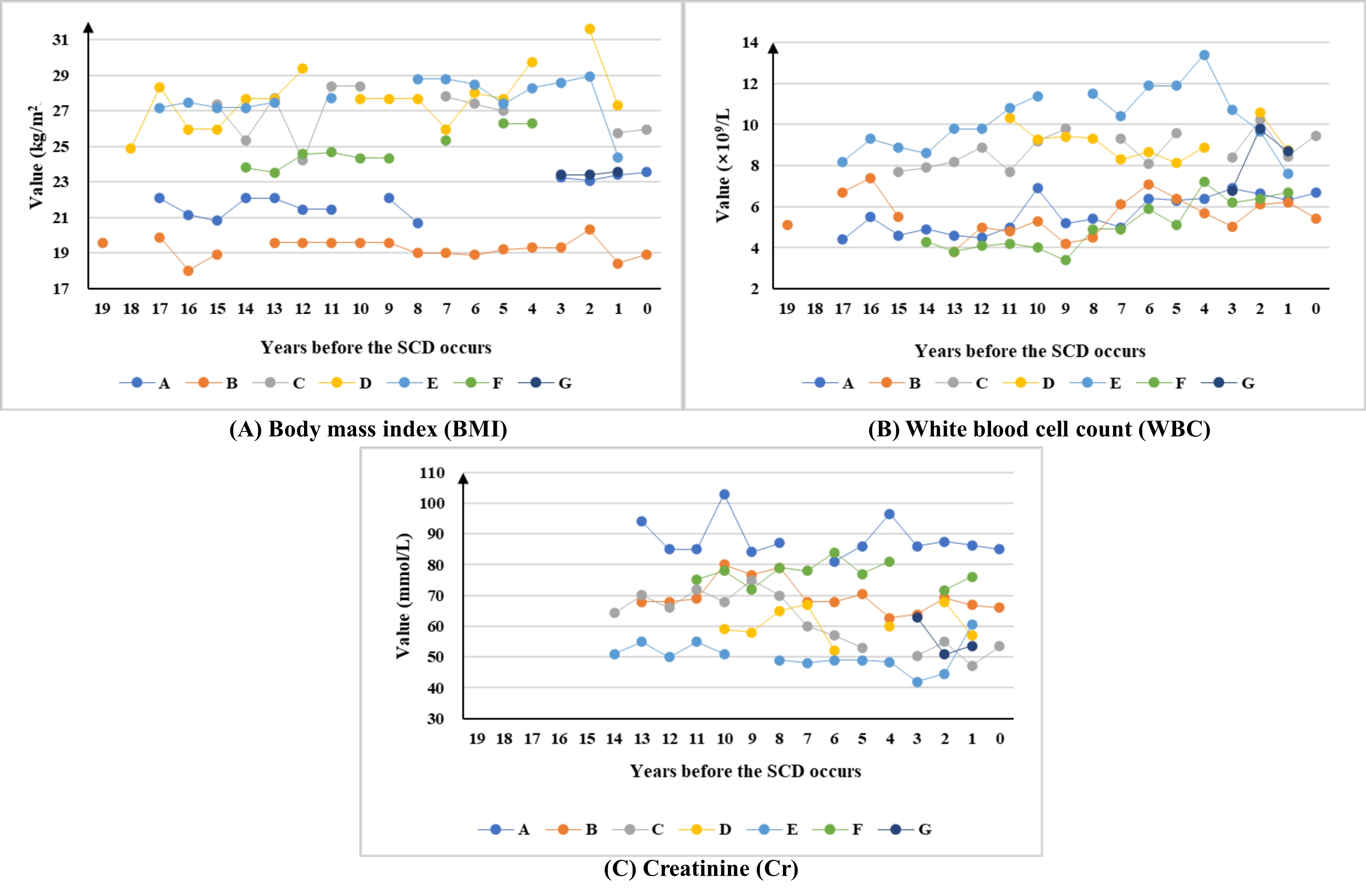  **Supplementary Figure S4. The long-term trend changes of indicators** **with stable patterns** |
| --- |

| 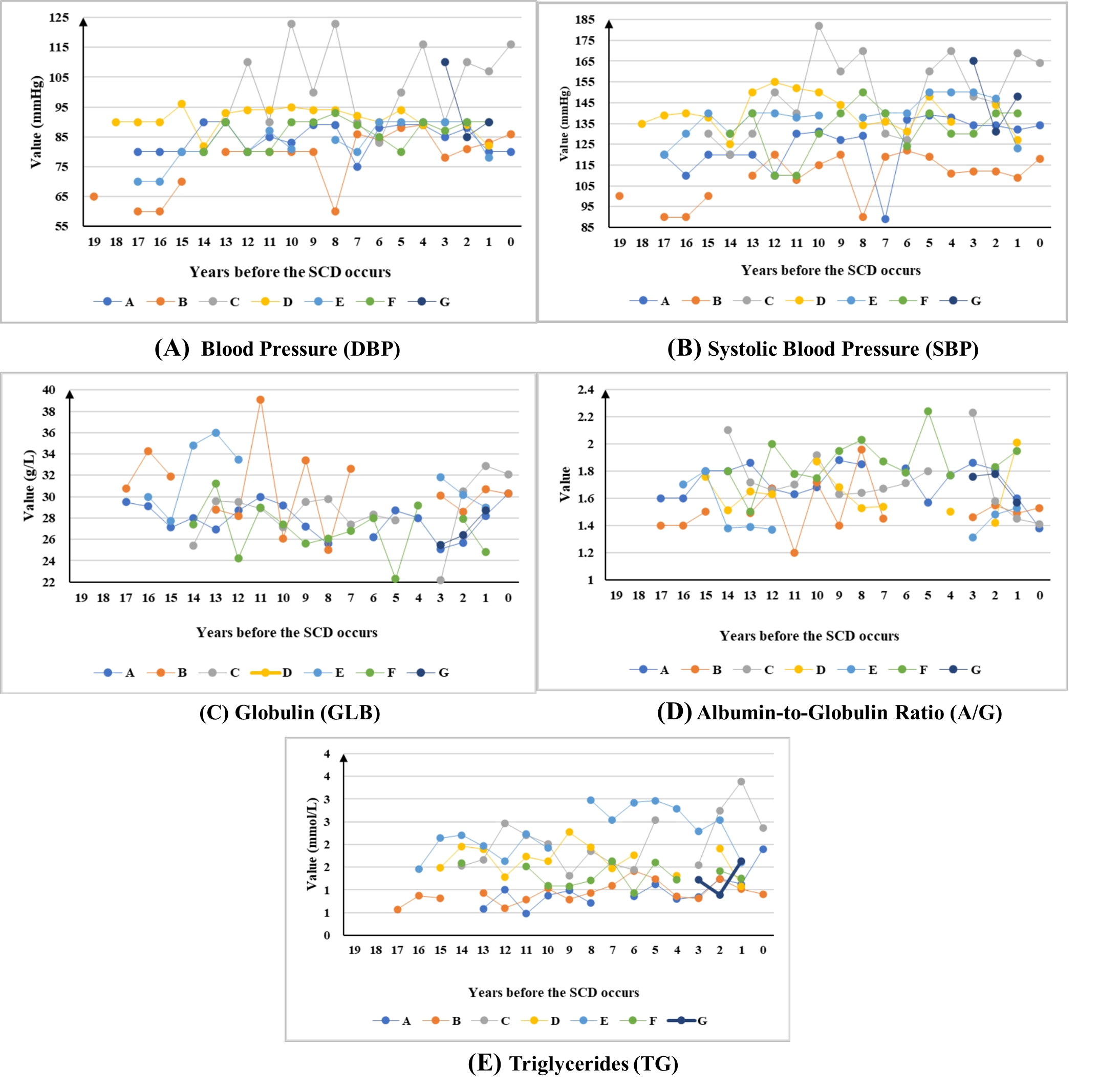  **Supplementary Figure S5. The long-term trend of indicators with no clear patterns** |
| --- |

On October 11, 2022, a worker on an offshore drilling platform suddenly experienced acute chest pain and subsequently experienced SCD during midday operations. The platform medical team promptly initiated the TMAS protocol, utilizing a coordinated sea‒land‒air rescue effort to rush the patient to a hospital on land. From symptom onset to definitive hospital treatment, the entire process took just over two hours, covering a distance exceeding 35 nautical miles. Ultimately, the patient's life was successfully saved.

The detailed emergency response procedures for this case were as follows (Figure 2):

(1) Discovery and reporting (11:00 AM)

The offshore worker experienced SCD on the platform. Coworkers immediately initiated emergency protocols and alerted the platform doctor.

(2) Initial treatment (11:00‒11:35 AM)

The platform doctor arrived within minutes to perform CPR and administer emergency medications. An ECG was conducted, revealing signs of acute myocardial infarction.

(3) Telemedicine information transmission (11:35 AM)

The platform doctor transmitted critical medical data, including ECG readings and patient status, to the onshore TMAS center and hospital via satellite telemedicine system.

(4) TMAS coordination (11:40 AM)

The onshore TMAS center immediately convened a multidisciplinary consultation with cardiology specialists, while simultaneously activating the TMAS system.

(5) Aerial rescue preparation (11:40‒12:25 AM)

The helicopter operator secured airspace clearance while the flight crew performed preflight checks and loaded medical equipment, concurrently with the receiving hospital preparing the landing zone and emergency teams for patient arrival.

(6) Takeoff from nearest onshore helipad (12:25 PM)

The rescue helicopter departed from the nearest coastal medical facility, initiating the aerial transport phase.

(7) Flight process (12:25‒12:42 PM)

The helicopter maintained strict adherence to its preapproved flight trajectory throughout the mission, with continuous airspace monitoring conducted by the TMAS command center. Simultaneously, the offshore platform doctor delivered uninterrupted critical care, including continuous physiological monitoring and necessary therapeutic interventions, while maintaining real-time telemedical consultation with land-based specialist physicians via the satellite telemedicine system.

(8) Platform arrival and patient transport (12:42 PM)

The helicopter arrived at the offshore platform and conducted a handover with the platform's medical team.

(9) Takeoff from offshore platform (1:01 PM)

The helicopter took off from the offshore platform and transported the worker to a land-based hospital, with monitored care under telemedicine guidance.

(10) Patient transport (1:20 PM)

The helicopter landed safely on the hospital helipad, where a prepositioned medical team and ambulance were standing. The worker was immediately transferred to the emergency room for treatment.

(11) Hospitalization and treatment (1:45 PM)

The worker was transported to the interventional cardiology center, where coronary angiography revealed right coronary artery occlusion. Following immediate stent implantation, the patient was admitted to the intensive care unit for postprocedural monitoring.
